# Parallel latent trajectories of mental health and employment earnings among 16- to 20-year-olds entering the US labor force: A 20-year longitudinal study

**DOI:** 10.1192/j.eurpsy.2022.865

**Published:** 2022-09-01

**Authors:** K. Dobson, S. Vigod, C. Mustard, P. Smith

**Affiliations:** 1 University of Toronto, Division Of Epidemiology, Dalla Lana School Of Public Health, Toronto, Canada; 2 Institute for Work & Health, Research, Toronto, Canada; 3 University of Toronto, Department Of Psychiatry, Toronto, Canada; 4 Women’s College Hospital and Research Institute, Department Of Psychiatry, Toronto, Canada

**Keywords:** MHI-5, latent parallel trajectory analysis, Depression, Anxiety, Psychological Distress, employment earnings

## Abstract

**Introduction:**

Depression and anxiety-related mental health and employment earnings are complexly intertwined but have rarely been studied as parallel processes.

**Objectives:**

Determine the number of latent parallel trajectories of mental health and employment earnings over two decades among a cohort of American youth entering the labor force, and estimate the association between baseline sociodemographic/health factors and latent trajectory class membership.

**Methods:**

This study included 8,173 participants from the American National Longitudinal Survey of Youth 1997, who were 13–17 years old in 1997. The survey occurred annually until 2011 then biennially until 2017. Mental health was measured eight times using the Mental Health Inventory-5 between 2000–2017. Employment earnings were measured annually between 1998–2017, where participants were 33–37 years old. Latent parallel trajectories were estimated using latent growth modeling. The association between baseline predictors and trajectory membership was explored using multinomial logistic regression.

**Results:**

Four latent trajectory classes were identified: good mental health, high earnings (3% of sample, average 2017 earnings ˜$196,000 USD); good mental health, medium earnings (23%, average 2017 earnings ˜$78,100); good mental health, low earnings (50%, average 2017 earnings ˜$39,500); and poor mental, low earnings (24%, average 2017 earnings ˜$32,000). Multinomial models revealed participants who were younger, female, Black, Hispanic, who had lower socioeconomic status, and had used marijuana at baseline had higher odds of belonging to the poor mental health, low earnings class.

**Conclusions:**

Findings highlight the stagnated, parallel course of poor mental health and earnings, and the influence of gender, race, adolescent socioeconomic status, and health behaviors on these trajectories.

**Figure:**
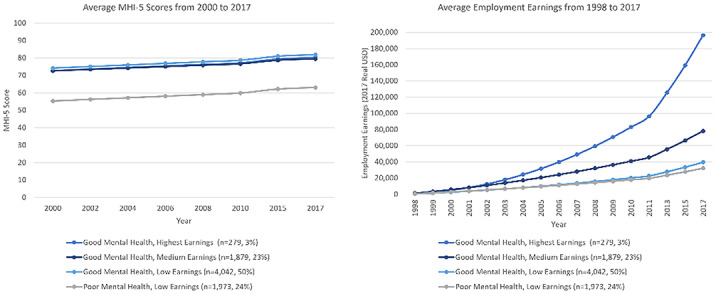

**Disclosure:**

No significant relationships.

